# The Interaction of Genetic Background and Mutational Effects in Regulation of Mouse Craniofacial Shape

**DOI:** 10.1534/g3.117.040659

**Published:** 2017-03-08

**Authors:** Christopher J. Percival, Pauline Marangoni, Vagan Tapaltsyan, Ophir Klein, Benedikt Hallgrímsson

**Affiliations:** * Alberta Children’s Hospital Institute for Child and Maternal Health, University of Calgary, Alberta T2N 4N1, Canada; † The McCaig Bone and Joint Institute, University of Calgary, Alberta T2N 4Z6, Canada; ‡ Department of Cell Biology and Anatomy, University of Calgary, Alberta T2N 4N1, Canada; § Department of Orofacial Sciences, University of California, San Francisco, California 94143; ** Program in Craniofacial Biology, University of California, San Francisco, California 94143; †† Department of Preventive and Restorative Dental Sciences, University of California, San Francisco, California 94143; ‡‡ Department of Pediatrics, University of California, San Francisco, California 94143; §§ Institute for Human Genetics, University of California, San Francisco, California 94143

**Keywords:** background effect, epistasis, skull, morphometrics, Sprouty, inbred background

## Abstract

Inbred genetic background significantly influences the expression of phenotypes associated with known genetic perturbations and can underlie variation in disease severity between individuals with the same mutation. However, the effect of epistatic interactions on the development of complex traits, such as craniofacial morphology, is poorly understood. Here, we investigated the effect of three inbred backgrounds (129X1/SvJ, C57BL/6J, and FVB/NJ) on the expression of craniofacial dysmorphology in mice (*Mus musculus*) with loss of function in three members of the Sprouty family of growth factor negative regulators (*Spry1*, *Spry2*, or *Spry4*) in order to explore the impact of epistatic interactions on skull morphology. We found that the interaction of inbred background and the Sprouty genotype explains as much craniofacial shape variation as the Sprouty genotype alone. The most severely affected genotypes display a relatively short and wide skull, a rounded cranial vault, and a more highly angled inferior profile. Our results suggest that the FVB background is more resilient to Sprouty loss of function than either C57 or 129, and that *Spry4* loss is generally less severe than loss of *Spry1* or *Spry2*. While the specific modifier genes responsible for these significant background effects remain unknown, our results highlight the value of intercrossing mice of multiple inbred backgrounds to identify the genes and developmental interactions that modulate the severity of craniofacial dysmorphology. Our quantitative results represent an important first step toward elucidating genetic interactions underlying variation in robustness to known genetic perturbations in mice.

Work over the past several decades has demonstrated that a large number of genes contribute to variation in complex traits such as stature or facial shape (Leamy *et al.* 1999; Wood *et al.* 2014). However, even as more contributing genes are identified, much of the phenotypic variance of complex traits remains unexplained. One reason for this observation is that genes exert effects that are highly context-dependent, creating epistatic interaction effects that account for significant portions of heritability (Mackay 2014). Craniofacial morphology is one such complex trait for which genetic interaction effects likely make important contributions to craniofacial variation. By investigating these epistatic interactions, we can obtain a deeper understanding of how they contribute to adult phenotypes, including normal craniofacial variation and the severity of craniofacial dysmorphology. Here, we quantify the influence of null mutations of Sprouty (*Spry*) 1 (Ensembl: ENSMUSG00000037211), 2 (Ensembl: ENSMUSG00000022114), and 4 (Ensembl: ENSMUSG00000024427) genes on adult mouse craniofacial morphology and use these genes as a model to quantify how three inbred genetic backgrounds (129X1/SvJ (129) RRID:IMSR_JAX:000691, C57BL/6J (C57) RRID:IMSR_JAX:000664, and FVB/NJ (FVB) RRID:IMSR_JAX:001800) modulate the morphological effects of known genetic perturbations.

Sprouty genes are negative regulators of a variety of growth factors, including FGF, VEGF, PDGF, and NGF. These genes have frequently overlapping expression domains (Minowada *et al.* 1999; Zhang *et al.* 2001) and partially redundant function (Taniguchi *et al.* 2007; Yang *et al.* 2010). In mice, Sprouty genes are known to regulate the development of the lung (de Maximy *et al.* 1999; Perl *et al.* 2003), ear (Shim *et al.* 2005), temporo-mandibular joint (Purcell *et al.* 2012), taste papillae (Petersen *et al.* 2011), kidney (Basson *et al.* 2005), and teeth (Klein *et al.* 2006, 2008; Lagronova-Churava *et al.* 2013; Marangoni *et al.* 2015), among other organs and structures [see Guy *et al.* (2009)]. These genes are also known to play critical roles during the ossification of limbs (Minowada *et al.* 1999; Taniguchi *et al.* 2007) and the craniofacial complex (Goodnough *et al.* 2007; Taniguchi *et al.* 2007; Welsh *et al.* 2007; Yang *et al.* 2010). However, little is known about how craniofacial features vary with changes in Sprouty gene expression. Overexpression of *Spry2* at early stages of facial development or induced neural crest-derived cell expression of *Spry1* can lead to midfacial hypoplasia and facial clefting, among other phenotypes (Goodnough *et al.* 2007; Yang *et al.* 2010). *Spry4*^−/−^ mice display reduced overall body size and mandibular defects, while *Spry2^−/−^*; *Spry4^−/−^* mice display embryonic lethality as well as alobar brains and cyclopia (Taniguchi *et al.* 2007).

Genetic background can influence the nature, penetrance, and severity of the phenotypic effect of a mutation (Doetschman 2009). Such differences result from variation of epistatic interactions, which may contribute significantly to apparent missing heritability for complex traits (Mackay 2014). In natural populations, interaction effects are very difficult to detect because of the statistical power required. For this reason, comparing the effects of the same mutation on different inbred backgrounds is an important strategy for identifying modifier genes within major gene networks that modulate the severity of disease phenotypes. Furthermore, when background effects are large, studying the effects of a mutation on multiple genetic backgrounds and their hybrids is required to assess the breadth of that mutation’s effect on phenotype (Linder 2006). Studying mice with a specific gene deletion on multiple inbred backgrounds with known genome-wide sequence variation will aid in the identification of genes with epistatic effects in specific developmental pathways, such as those underlying variation in the craniofacial complex.

Genetic background effects for craniofacial dysmorphology are known (*e.g.*, Quinn *et al.* 1997; Hide *et al.* 2002; Dixon and Dixon 2004; Fairbridge *et al.* 2010). However, previous studies have not systematically quantified modulation of phenotype for mutations in the same pathway on different backgrounds. Geometric morphometric methods quantify skeletal morphology to reveal subtle yet significant changes in size and shape. We report here that Sprouty null mutations cause significant modifications in overall craniofacial size and shape. However, the specific effects of each mutation also depend significantly on genetic background. These background effects include changes in both the magnitude and nature (direction) of craniofacial dysmorphology. The magnitudes of these interaction effects are large, accounting for nearly as much variation as the average mutational effects across backgrounds.

## Materials and Methods

All mice (*Mus musculus*) were bred at the University of California, San Francisco in compliance with relevant animal care guidelines and experimentation protocols. Mouse lines carrying null alleles of *Spry1* (Basson *et al.* 2005), *Spry2* (Shim *et al.* 2005), and *Spry4* (Klein *et al.* 2006) were acquired as knockout mice (gift from G. Martin) and maintained on a Crl:CD1(ICR) mixed background within the Klein lab. These three strains were then independently backcrossed, using the standard procedure for producing congenic lines, onto the 129X1/SvJ, C57BL/6J, and FVB/NJ backgrounds for between 6 and 27 generations (See Supplemental Material, File S3 for specimen generation numbers). The Sprouty genotype of each backcrossed specimen was assessed by PCR. Heterozygotes of these backcrossed null mice were crossed to produce litters that included homozygote null (−/−), heterozygote null (+/−), and homozygote controls (+/+) of each knockout by background pair. Therefore, our sample includes representatives from 18 genotype-by-background combinations (Table 1). While some of our sample were not strictly isogenic in their genetic background (<20 generations of backcrossing), congenic backcrossing for six generations results in >95% homozygosity across the genome (Silver 1995). All specimens were killed at ∼8 wk of age and stored at −20°. Compared to other model organisms, the sample sizes of this selection of mutant mice might be considered modest, particularly when compared to the number of landmark coordinates measured. We try to address this issue by completing many of our statistical tests on summary scores or after dimension reduction. Additionally, we rely on nonparametric methods for determining significance, which are less constrained by the number of phenotypic variables (Collyer *et al.* 2015).

**Table 1:** Sample sizes

	129	C57	FVB
*Spry1^+/+^*	10	9	19
*Spry1^+/−^*	10	10	20
*Spry1^−/−^*	19	10	10
*Spry2^+/+^*	17	10	13
*Spry2^+/−^*	14	19	28
*Spry2^−/−^*	9	13	9
*Spry4^+/+^*	6	19	18
*Spry4^+/−^*	11	18	20
*Spry4^−/−^*	16	8	13

Sample sizes of all inbred background (listed above) and Sprouty genotype combinations (listed on the left).

Three-dimensional coordinates of 54 previously defined (Percival *et al.* 2016) adult landmarks were identified on skull surfaces derived from microcomputed tomography images of specimen heads (0.035 mm^3^ voxel size). We completed two general categories of morphometric analysis: (1) Euclidian Distance Matrix Analysis (EDMA) [see Lele and Richtsmeier (2001)], which allows identification of raw differences in unscaled form (the combination of shape and size) between groups and (2) Procrustes superimposition-based Geometric Morphometrics [see Zelditch *et al.* (2012)], which allow the comparison of broader patterns of shape variation between groups after removing differences in scale (Hallgrimsson *et al.* 2015). EDMA analyses were completed with custom functions in R (R Developmental Core Team 2008), which were based on published formulas (Lele and Richtsmeier 2001) and R code (Hu 2007). Procrustes superimposition, calculation of centroid sizes, and Procrustes ANOVA were performed using the geomorph package (Adams and Otárola-Castillo 2013; Adams *et al.* 2016) in R.

Significant differences in mean centroid size between homozygote knockout specimens and their littermate controls were identified by comparing knockout mean centroid sizes against the 95% C.I. of littermate control centroid size, produced with bootstrap tests (1000 permutations). EDMA FORM analysis was used to identify linear distances that significantly differ between homozygote knockouts and their littermate controls (α = 0.05). ANOVA of Procrustes superimposed coordinates was completed for all specimens in our sample, with genotype (*e.g.*, *Spry1*^+/−^), inbred background (*e.g.*, C57), and the interaction between genotype and inbred background as covariates, using geomorph’s Procrustes ANOVA function. The randomized residual permutation procedure (Collyer *et al.* 2015) was used to determine significance of factor effects. Estimated Rsq values are coefficients of determination for each term, which are interpreted as the percentage of landmark coordinate variance accounted for by a given factor. This analysis was used to determine whether genotype and background contribute significantly to adult skull shape and how much of the variance in skull shape they are associated with. In addition, Procrustes distances between mean group shapes quantified the strength of overall shape differences, while a bootstrapping algorithm indicated the significance of those shape differences (α = 0.05).

Pairwise correlations between vectors of shape change quantified whether the direction of skull shape change is similar when a given null mutation is found on different backgrounds. Vectors were calculated as mean differences in principal component scores between +/+ and −/− genotypes on a single background, similar to how they are calculated for vector correlations in trajectory.analysis() within geomorph (Collyer and Adams 2013). Permutations (*n* = 1000) of vector correlations calculated from four groups of 20 random specimens were used to produce a baseline 95% C.I. to determine whether the pairwise vector correlations in our sample were significantly different to zero.

### Data availability

A more detailed *Materials and Methods* description is available within File S1. Mouse strains are available upon request. All data necessary to replicate our analyses are available as supporting files. File S2 is a .csv tabular file of skull landmark coordinates where each row is a specimen and each column is a landmark coordinate, where the three-dimensional coordinates of the first landmark are followed by the three-dimensional coordinates of the second, then the third, etc. Landmark coordinate names [defined in Percival *et al.* (2016)] are found in the first row and specimen identification numbers are found in the first column. All landmark coordinate values are in mm. File S3 is a classifier and covariate .csv table where each row is a specimen and the columns list specimen identification, sex, genotype, inbred background, genotype and background, weight in grams, and backcross generation number (*N*). File S4 includes tables that further detail the PCR primers used to genotype our Sprouty null mice. Microcomputed tomography images of all specimen heads can be found on FaceBase (https://www.facebase.org), linked to the principal investigators (B.H. and O.K.).

## Results

### Effect on craniofacial size

**Figure 1:**
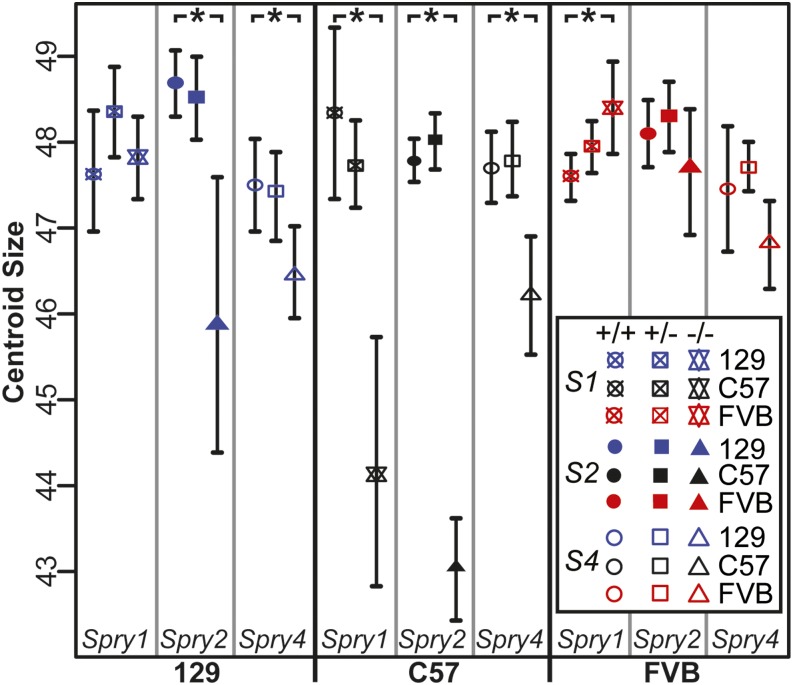
Centroid size C.I. Mean centroid size for each group of specimens and surrounding 95% C.I.-estimated via bootstrap (1000 permutations). Groups are clustered by inbred genetic background, then Sprouty genotype, with homozygote controls, heterozygotes, and homozygote null littermates from left to right. Asterisk (*) indicates a significant difference in centroid size between +/+ and −/− of a genotype/background combination, based on +/+ C.I.

To quantify the effect of Sprouty gene loss on craniofacial size within all littermate groups, centroid sizes were compared between homozygote knockout and controls with bootstrap tests. Six *−/−* centroid size means were different to littermate *+/+* centroid sizes, based on *+/+* 95% C.I. These included significant skull size reduction for all three Sprouty gene deletions on the C57 background, reductions for *Spry2^−/−^* and *Spry4^−/−^* on the 129 background, and a significant increase in size for *Spry1^−/−^* on the FVB background (Figure 1).

While the overall centroid size of a knockout skull may have been reduced, all dimensions of the skull were not necessarily reduced to the same extent or even reduced at all. We completed an EDMA FORM analysis on each pair of homozygote knockouts and littermate controls, which produces a control:knockout ratio for each linear distance between our landmarks. A permutation test was completed to determine whether each individual linear distance ratio is significantly different from one.

**Figure 2:**
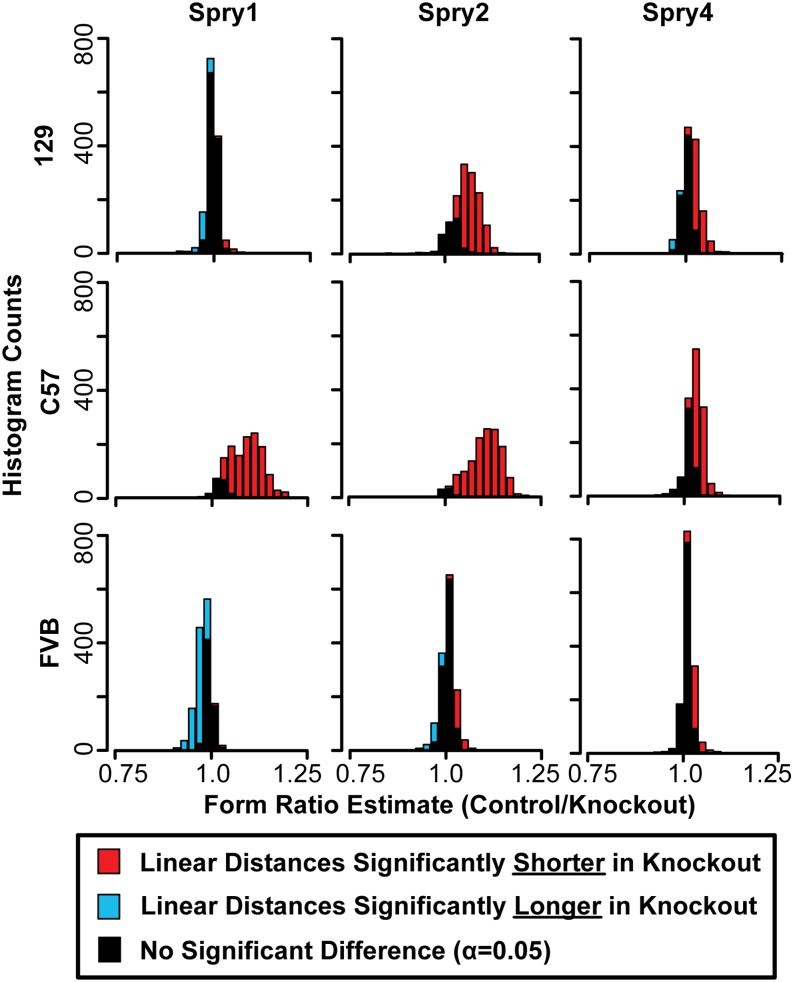
Form ratio histograms. Histograms of the ratios of all linear distances between our landmarks, where the mean linear distance value of the control mice (+/+) is the numerator and the mean linear distance value of the homozygous null mice (−/−) is the denominator. The proportion of linear distance ratios that are significantly different to one at α = 0.05, based on bootstrapping the control group, are indicated by colors in the histogram bars. Ratio estimates >1 indicate that the linear distance is shorter in −/− mice than in +/+ mice (red), while estimates <1 indicate that the linear distance is longer in −/− mice than in +/+ mice (blue).

While a Procrustes-based shape analysis can indicate that a genotype has a relatively small vault compared to the face, it cannot distinguish whether this pattern is due to increased face size or decreased vault size. Because EDMA FORM analysis compares the raw lengths of all linear distances on the head, it can help illustrate how much of the skull and which aspects of the skull display significant size change. All pairwise FORM comparisons between +/+ and −/− littermates displayed some significant differences in linear distances between knockouts and controls (Figure 2). A ratio of one indicates that the length of a linear distance is equivalent for both genotypes. Ratios further from one indicate more pronounced effects on raw linear distance length. Ratios were plotted as histograms, providing a visualization of general direction and strength of Sprouty null mutations on linear dimensions of skull size. The strongest Sprouty knockout effects led to a significant reduction to many linear distances, with ratios being distributed across a wider range of ratio values. Stronger effects were noted for the same genotypes for which overall centroid size was significantly different between homozygote deletion and homozygote control specimens.

### Genetic basis for craniofacial shape variation

Comparisons of craniofacial shape were completed after Procrustes superimposition of landmarks to standardize scale and orientation of all specimens. An ANOVA analysis on the Procrustes superimposed landmark coordinates indicated that inbred background and Sprouty deletion genotype, as well as the interaction of these two factors, were each significant contributors to craniofacial shape variation. Background accounted for >20% of the total craniofacial shape variation (Rsq), while knockout genotype and the interaction of background and genotype each accounted for ∼11% of the shape variation. This means that, after accounting for background and genotype identity, the background by genotype interaction accounted for as much of the Procrustes coordinate variation as genotype alone (Table 2).

**Table 2:** Procrustes ANOVA

	Df	SS	MS	Rsq	F	Z	Pr (> F)
Inbred background	2	0.111	0.055	0.232	76.01	25.28	0.001*^a^*
Sprouty genotype	8	0.054	0.007	0.114	9.29	6.74	0.001*^a^*
Background:genotype interaction	16	0.056	0.003	0.117	4.78	4.16	0.001*^a^*
Residuals	351	0.256	0.001				
Total	377	0.476					

The results of an ANOVA analysis of Procrustes coordinates including the factors of (1) inbred background, (2) sprouty genotype, and (3) the interaction of inbred background and Sprouty genotype. Df, degrees of freedom; SS, sum of squares; MS, mean square; Rsq, total craniofacial shape variation; F, F-statistic; Z, Z-score; Pr (>F), p-value .

a Indicates significance of the factor effect at α = 0.001 or lower.

### Background shape differences

**Figure 3:**
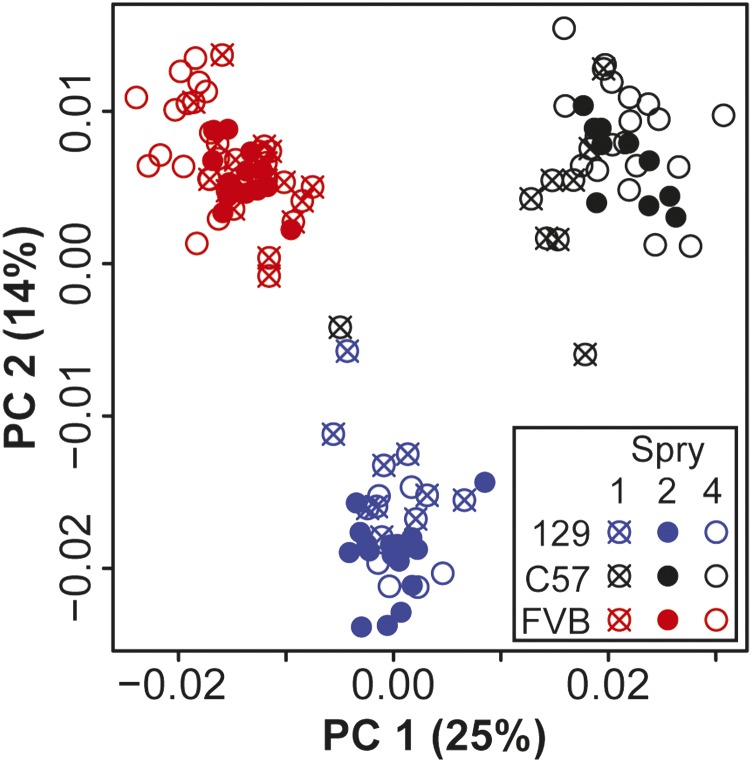
Control shape PCA. Principal component (PC) 1 and PC2 from a PCA of control (+/+) specimen landmark coordinates after Procrustes superimposition (including scaling of landmark configurations), but without any allometric regression or background correction. The C57 specimen that plots close to 129 control specimens does not represent a plotting or background identification error. PCA, principal component analysis; %, % of variance.

Inbred background identity explains >20% of craniofacial shape variation (Table 2). Procrustes distances, calculated between the mean Procrustes coordinates of all control specimens on one inbred background and the mean coordinates for all control specimens on another inbred background, quantified the degree of shape difference between inbred backgrounds. Whether these distances were significantly different to zero was determined with permutation tests for each genotype pair. The control shape of each background was significantly different to the control shape of the other two backgrounds and all background mean shapes were approximately the same Procrustes distance apart (Table 3). There was strong separation between inbred backgrounds along principal components one and two within a PCA of the Procrustes coordinates of all control specimens (Figure 3).

**Table 3:** Procrustes distance test of mean shapes of controls from different inbred backgrounds

	129	C57	FVB
129	0		
C57	0.21*^a^*	0	
FVB	0.20*^a^*	0.24*^a^*	0

Procrustes distances between mean shapes of controls from different inbred backgrounds of specific Sprouty null mutation and inbred background combinations.

a Differences are significant (α = 0.05).

**Figure 4:**
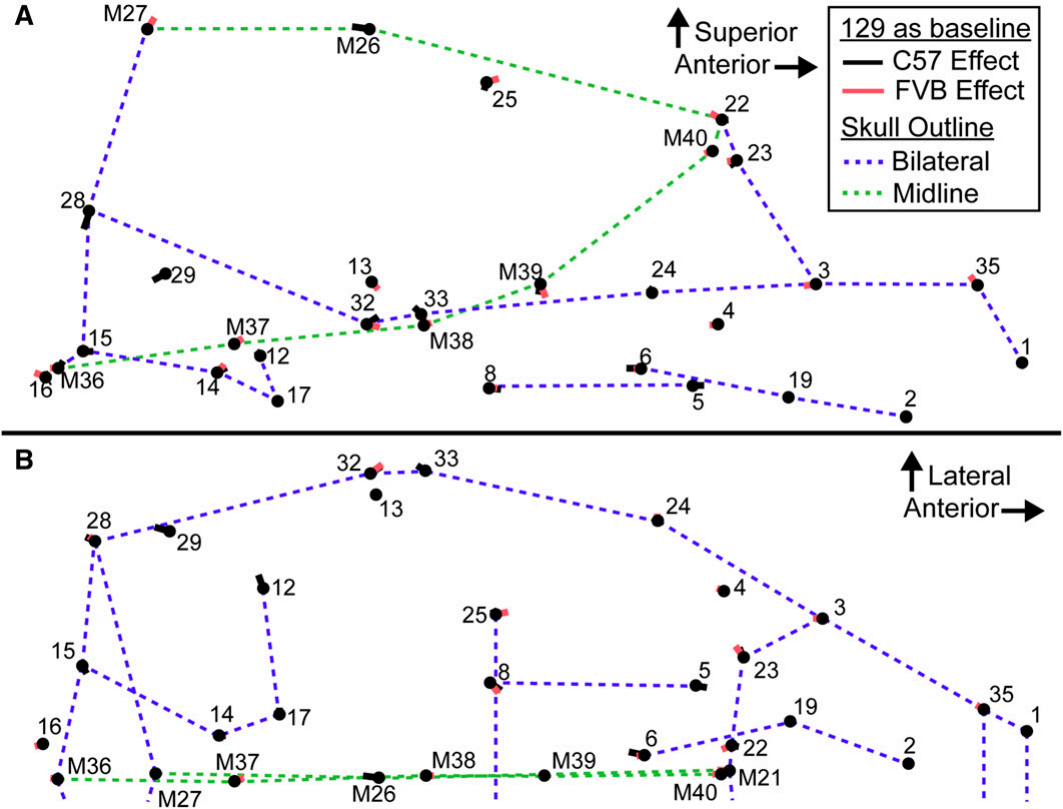
Background control shape difference vectors. Vectors illustrating the difference in mean shape between the three backgrounds from the (A) lateral and (B) superior views (only midline and left side landmarks shown). In both cases, the nose of the mouse is to the right. 129 is used as a baseline for comparison, the black vectors go from 129 mean shape to C57 mean shape (vector length is scaled 1:1 with landmark coordinates), the red vectors go from 129 mean shape to FVB mean shape. Thin skull outlines are references for sagittal midline features (green) and bilateral features (blue), which are meant to provide a frame of reference within the skull. Landmark numbers match a previously defined landmark list (Percival *et al.* 2016) and midline landmarks are highlighted with a preceding “M.” This is not a representation of the covariation associated with a principal component, but is the actual vector of difference in mean Procrustes coordinates between these groups.

We plotted the difference between mean control background shapes after Procrustes superimposition (Figure 4). These vectors must be interpreted with care, as they describe differences in shape after scale is removed by Procrustes superimposition. Therefore, all differences in length, width, or height described in the following “shape effect” sections are descriptions of relative shape changes and not absolute differences in length, width, and height. It should also be noted that an absolute decrease in skull length or an absolute increase in skull width can both lead to identical shape vectors that suggest a relatively short skull. Combining information from analysis of size variables (above) and shape variables (below) is necessary to make the distinction between these two possibilities.

The C57 and FVB backgrounds were each compared to the 129 background, although any of the backgrounds could have served as a baseline. C57 controls differed in shape from 129 controls largely in posterior palate, petrous temporal, and midline vault shape (Figure 4, black lines). The relative position of facial and anterior palate landmarks did not differ substantially, although the posterior end of the palatal foramen was further posterior (LM 6) and the molar row (LMs 5 and 8) was relatively anterior and medial in the C57 background. The C57 petrous temporal (LMs 12, 14, 15, 17, and 29) was rotated clockwise if viewed from the superior aspect, meaning that the anterior portion was rotated laterally, the lateral portion was rotated posteriorly, and the posterior portion was rotated medially. The lateral edges of the cranial vault were found to be relatively inferior (LMs 23, 25 and 28), while the frontal bone was relatively long when compared to parietal, with a posterior coronal suture (LM M26) and an anterior frontonasal suture (LMs M21 and 22) in C57. The zygomatic arch (LMs 24, 32 and 33) was also relatively superior (Figure 4, black lines).

FVB controls differed from 129 controls largely in cranial vault and midline cranial base shape (Figure 4, red lines). The posterior end of the molar tooth row (LM 8) was relatively medial and anterior in FVB. The maxillary portion of the zygomatic arch (LMs 3 and 23) and the frontonasal suture (LMs M21 and 22) were relatively posterior in FVB, leading to a relatively short frontal bone. The midline point at the intersection of the sagittal and lambdoid sutures was superior (LM M27), indicating a relatively high posterior cranial vault height. The posterior portion of the zygomatic arch (LMs 32 and 33) was relatively inferior. The basioccipital was relatively longer in FVB mice, due to a more posterior foramen magnum border (LM M36) and a more anterior basisphenoid synchondrosis (LM M37). The cranial base also included a relatively anterior prespheno–sphenoid synchondrosis (LM M38) and a more inferior border between the presphenoid and the ethmoid (LM M39), which led to a larger cranial base angle (Figure 4, red lines).

### Shape effect of homozygous deletion

Procrustes distances, calculated between the mean Procrustes coordinates of homozygous knockout and control littermate groups, represent the degree of shape difference caused by Sprouty gene loss. Permutation tests for each genotype pair were used to test whether these distances were significantly different to zero. The magnitude of the craniofacial shape effect of Sprouty deletions differed by gene and by inbred background (Table 4). The Procrustes distances between the mean shapes of *Spry1^+/+^* and *Spry1^−/−^* on the C57 background, *Spry2^+/+^* and *Spry2^−/−^* on the 129 background, and *Spry2^+/+^* and *Spry2^−/−^* on the C57 background were each greater than the Procrustes distances between any two inbred background strains. Therefore, these combinations of Sprouty gene deletion and inbred background lead to greater differences in skull shape within a single background than the typical shape differences between controls from two inbred backgrounds.

**Table 4:** Procrustes distance test of mean shapes of control and homozygote null mutation genotypes

	Spry1	Spry2	Spry4
129	0.12*^a^*	0.28*^a^*	0.18*^a^*
C57	0.36*^a^*	0.34*^a^*	0.16*^a^*
FVB	0.15*^a^*	0.14*^a^*	0.12*^a^*

Procrustes distances between mean shapes of control and homozygote null mutation genotypes of specific Sprouty null mutation and inbred background combinations.

a Differences are significant (α = 0.05).

**Figure 5:**
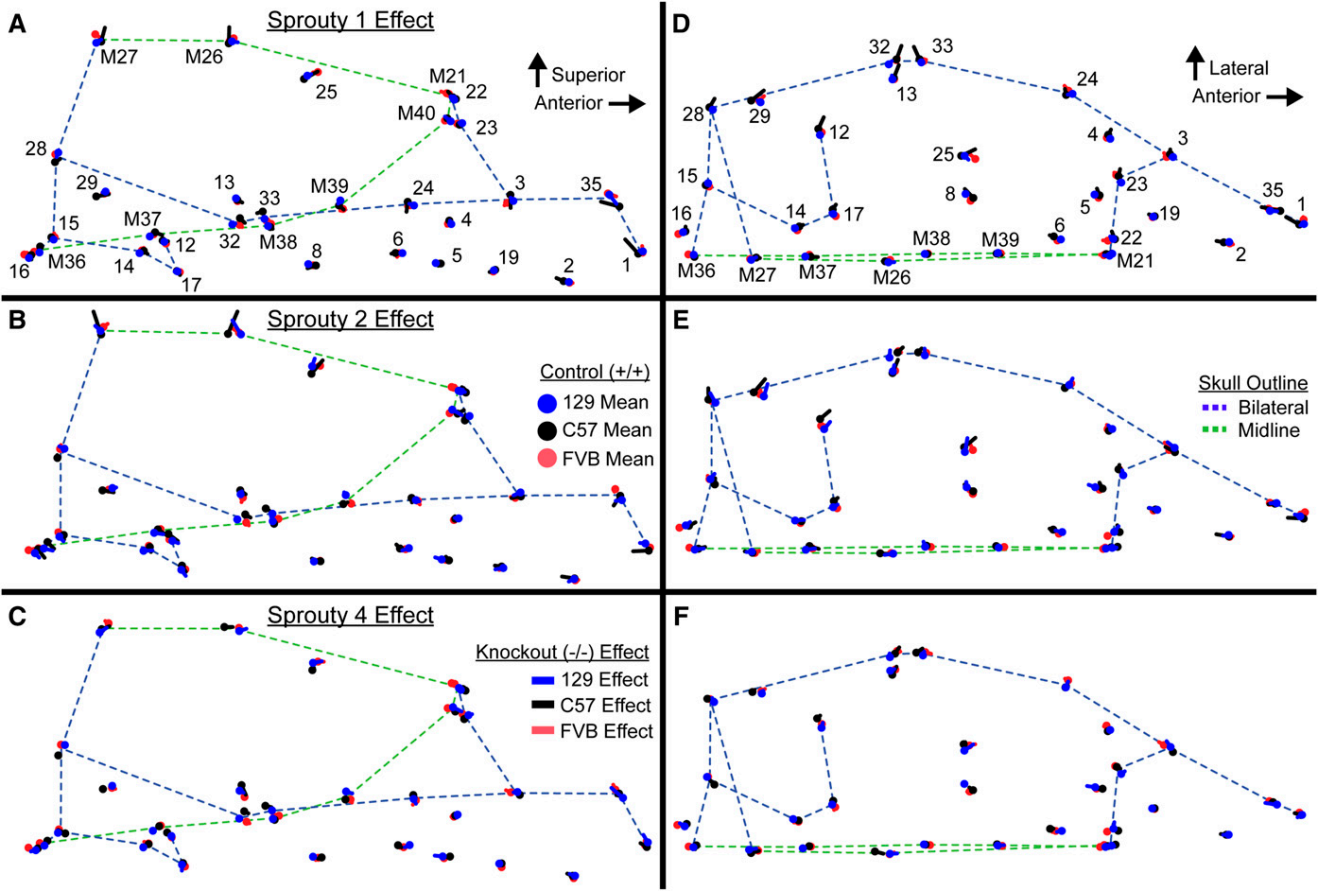
Sprouty shape effect vectors. Vectors illustrating the effect of homozygote Sprouty gene deletion from the (A–C) lateral view and the superior view (D–F). Only the left and midline points of the mouse skull are represented in the superior view panels. Points represent mean control shape, which is connected to mean homozygote knockout shape by a line. Blue represents mice on the 129 background, black on the C57 background, and red on the FVB background. Thin skull outlines are references for sagittal midline features (green) and bilateral features (blue), which are meant to provide a frame of reference within the skull. Landmark numbers match a previously defined landmark list (Percival *et al.* 2016) and midline landmarks are highlighted with a preceding “M.” This is not a representation of the covariation associated with a principal component, but is the actual vector of difference in mean Procrustes coordinates between these groups.

While it is clear that deleting the same gene on multiple backgrounds leads to varying severity of shape changes, it is possible that the direction of shape effects associated with a given gene knockout is similar between backgrounds. Vector representations of the landmark specific shape effects associated with homozygous Sprouty deletion allow us to compare the influence of background across the skull (Figure 5). Furthermore, correlations between summary shape change vectors, calculated from principal component scores, quantify the overall similarity in the shape effects of given Sprouty null mutations across multiple backgrounds. We find that the direction of shape changes are significantly correlated for *Spry2* loss on 129 and C57 and on 129 and FVB, as well as for *Spry2* loss between 129 and C57 (Table 5). In addition, we note very high and significant shape change vector correlations between the three groups for which shape is most severely affected by gene loss. These three genotypes [*Spry1* loss on C57 (Figure 5, A and D black lines), *Spry2* loss on 129 (Figure 5, B and E blue lines), and *Spry2* loss on C57 backgrounds (Figure 5, B and E blue lines)], were each associated with a relatively tall vault (LMs M26 and M27), although the effect on the length of specific vault bones varies.

**Table 5:** Correlations of Sprouty null mutation shape effect vectors across backgrounds

	129 *vs.* C57	129 *vs.* FVB	C57 *vs.* FVB	129 *Spry2^−/−^* *vs.* C57 *Spry2^−/−^*	C57 *Spry1^−/−^* *vs.* C57 *Spry2^−/−^*	C57 *Spry1^−/−^* *vs.* 129 *Spry2^−/−^*
*Spry1^−/−^*	0.02	0.17	0.22			
*Spry2^−/−^*	0.72*^a^*	0.54*^a^*	0.42			
*Spry4^−/−^*	0.52*^a^*	0.42	0.19			
Large effects				0.72*^a^*	0.75*^a^*	0.65*^a^*

Correlations between vectors of shape change associated with specific Sprouty null mutations on different backgrounds and correlations between vectors of shape change associated with the genotype/background combinations with the strongest shape change effects.

a Correlations that are significantly higher than zero, based on a permutation-based 95% C.I. (−0.48 to 0.47).

**Figure 6:**
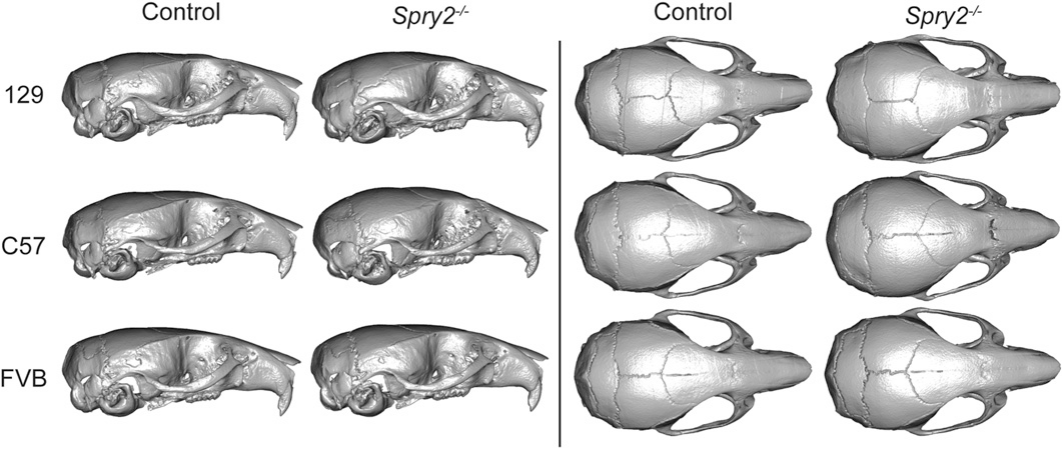
Skull surface visualizations. Snapshots of CT-derived skull surfaces of mean control specimens and *Spry2^−/−^* specimens from all three backgrounds, viewed from the lateral (left) and superior views (right). Each mean specimen (six in total) was created by identifying the CT-based skull surface of the specimen closest to the mean shape of inbred background controls or *Spry2^−/−^* groups and morphing this surface to the actual mean Procrustes coordinates for that background/genotype. These images represent shape variation, but not size variation (*i.e.*, images are not to scale). CT, computed tomography.

The relatively lateral position of lateral vault landmarks (LMs 23, 25 and 28) combined with increased vault height to produce a more rounded vault shape in these severely affected genotypes. Posterior cranial base landmarks (LMs 12 and 17, and M37) were also found in relatively lateral, anterior, and inferior positions, while anterior landmarks of the palate (LMs 2, 5, 6, and 19) were found more posteriorly in these knockout mice, leading to a relatively short cranial base. Overall, these three groups with the strongest deletion effects produced a change in shape leading to a relatively short and wide skull, with a rounded cranial vault, and an anterior-inferior cranial base combined with a posterior palate leading to a more highly angled inferior profile. Skull surface visualizations of the mean shape (after scaling) of inbred background controls and each *Spry2^−/−^* group also illustrated some of these differences (Figure 6). EDMA FORM comparisons of Sprouty homozygote control and null mice, where ratios of linear distances are produced from unscaled measurements, showed that posterior cranial vault widths were either not significantly reduced or were actually wider (not just relatively wider) in the homozygous knockout genotypes that display severe skull size reductions (Table 6). This indicated that even though much of the skull size was considerably reduced for these severe homozygote deletions, raw vault width was not significantly reduced.

**Table 6:** Vault bone length comparisons

	*Spry1* on C57			*Spry2* on 129			*Spry2* on C57		
	*+/+*	*−/−*	Ratio	*+/+*	*−/−*	Ratio	*+/+*	*−/−*	Ratio
Frontal length (M21–M26)	7.14 (0.31)	6.45 (0.58)	1.11*^a^*	7.00 (0.23)	7.03 (0.42)	1.00	7.41 (0.15)	6.50 (0.34)	1.14*^a^*
Parietal length (M26–M27)	3.96 (0.47)	3.52 (0.34)	1.12*^a^*	4.37 (0.19)	4.04 (0.47)	1.09*^a^*	3.88 (0.11)	3.96 (0.47)	0.99
Frontal and parietal length (M21–M27)	11.02 (0.39)	9.86 (0.75)	1.12*^a^*	11.29 (0.31)	10.91 (0.66)	1.04	11.21 (0.12)	10.33 (0.35)	1.09*^a^*
Vault width (R25–L25)	6.05 (0.14)	5.89 (0.14)	1.03*^a^*	5.90 (0.21)	6.31 (0.41)	0.94*^a^*	6.04 (0.09)	5.99 (0.29)	1.01

The first two columns of each section include mean raw lengths of midline vault linear distances (mm), followed by SDs in parentheses, for control (+/+) and homozygote null mutant (−/−) specimens of the three most severely affected background/genotype combinations. The third column in each section includes ratios of +/+ length over −/− length.

a Ratio value is significantly different from 1.0 (*P* = 0.05) based on an EDMA (Euclidian Distance Matrix Analysis) FORM difference permutation test.

### Midline vault length

Variation in the length of the midline vault and the relative contribution of various bones to overall vault length indicated that this would be an interesting region within which to quantify the effects of Sprouty gene expression. Overall, control specimens of the C57 background had significantly longer frontal bones and shorter parietal bones than control specimens on the 129 background, although there was no difference in total midline cranial vault length. This reversed pattern of midline bone contribution to total vault length was also seen for wild-type littermates that served as control specimens for *Spry2* and *Spry4* null mutants (Figure 5, M26 and M21, black and blue dots).

**Figure 7:**
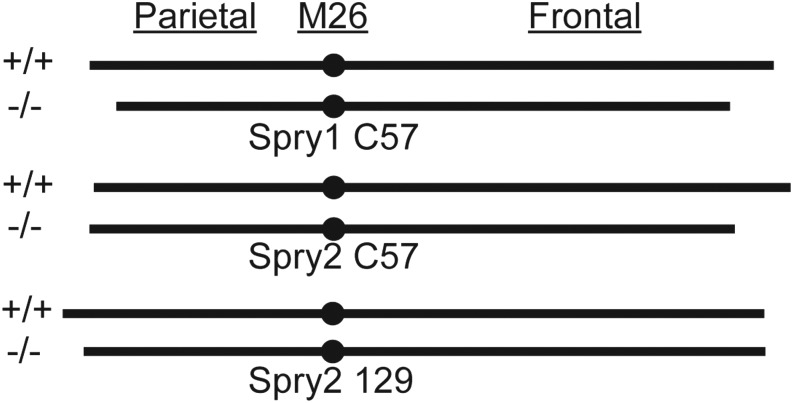
Mean vault bone length comparisons. A simplified illustration of the relative contribution of the frontal bone and the parietal bone to total vault length (see also Table 6) for controls and homozygote knockouts of *Spry1* on the C57 background (top), *Spry2* on C57 (middle), and *Spry2* on 129 (bottom). All illustrated bone lengths are to scale.

*Spry2^−/−^* mice on the C57 background displayed a significantly reduced midline vault length compared to controls, but this reduction appeared to be driven entirely by reduced frontal bone length. While *Spry2* loss on the 129 background did not lead to a statistically significant vault length reduction, there was a suggestive decrease in parietal bone length (Figure 7 and Table 6). This pattern suggested the vault bone that is relatively long on a given background might be the bone most strongly reduced by *Spry2* loss. Shape effect vectors, which are plotted after landmark coordinates are scaled, support this interpretation (Figure 5). Landmark M26 at the border between frontal and parietal bones was displaced anteriorly and superiorly in *Spry2^−/−^* mice on the C57 background, but it is displaced posteriorly and superiorly in *Spry2^−/−^* mice on the 129 background. Changes in midline vault length associated with Sprouty gene deletion illustrated the complexity of background influence on the effect of reduced Sprouty signaling.

## Discussion

We have quantified how inbred background modulates the effect of gene loss on craniofacial size and shape in mice. The interaction of Sprouty gene loss and inbred background explained as much craniofacial shape variation as Sprouty gene loss alone (Table 2). For example, while *Spry2^−/−^* mice displayed severe craniofacial size reduction and dysmorphology when on the 129 and C57 backgrounds, the *Spry2^−/−^* mice on the FVB background displayed much subtler phenotypic changes. These pronounced background effects indicate that certain background genotypes are more robust to variation in Sprouty gene expression. In addition to variation in the severity of craniofacial dysmorphology, background effects influenced the nature of that dysmorphology. For example, Sprouty null mice on both the 129 and C57 background displayed relatively rounded cranial vaults (Figure 5 and Figure 6), but this was associated with a significant reduction in frontal bone length only on the C57 background (Figure 7 and Table 6). While the specific modifier genes responsible for the background effects we quantified are currently unknown, our results illustrate intriguing patterns of background effect across three Sprouty null mutations. Our results also highlight the potential value of crossing mice of multiple backgrounds that share specific mutations in order to identify important epistatic interactions that underlie disease penetrance and severity.

Although genes associated with many diseases have been identified through genetic linkage analysis in humans, genetic heterogeneity within and between human populations can hinder efforts to replicate the results of these linkage studies (Chari and Dworkin 2013; Mackay 2014). Differences in dysmorphology between individuals with the same disease allele may be caused by variation in modifier genes, but significant heterogeneity within our species makes it difficult to identify these modifier genes. Inbred animal models are a powerful alternative for identifying modifier genes because a given disease mutation can be engineered into mice with uniform background (Linder 2006). Indeed, backcrossing or intercrossing multiple inbred lines with the same mutation allows direct mapping of phenotypic variation to genes interacting with a shared mutation (Le Corvoisier *et al.* 2003). Documenting the effects of such epistatic interactions that modulate the effect of gene loss or known mutations is an important step toward identifying the modifier genes responsible for such epistatic effects.

### Sprouty deletion effects

Severe craniofacial phenotypes occur in *Spry1^−/−^* mice on the C57 background, *Spry2^−/−^* mice on 129, or *Spry2^−/−^* mice on C57. We found that these mutants display the strongest size (Figure 1 and Figure 2) and shape effects (Table 4). This overlap may be partially explained by allometric variation that was still present in our Procrustes coordinate dataset, even though scale was removed during Procrustes superimposition. The severe shape effects include a relatively short and wide skull, a rounded cranial vault, and an anterior–inferior cranial base combined with a posterior palate leading to a more highly angled inferior profile. Our results indicated that loss of either *Spry1* or *Spry2* within certain genetic contexts leads to severe craniofacial dysmorphology with grossly similar characteristics (Figure 5 and Table 5). Overall, loss of *Spry4* had a less severe effect on craniofacial development, suggesting that mutations of *Spry4* are less likely to perturb craniofacial development than loss of either *Spry1* or *Spry2*. For example, the less severe dysmorphology of *Spry4^−/−^* mice on the C57 background may be related to the fact that *Spry1* and *Spry2* are typically expressed in embryonic facial epithelium, while *Spry4* is expressed in embryonic facial mesenchyme (Zhang *et al.* 2001). The overlapping expression patterns may indicate potential functional redundancy for *Spry1* and *Spry2* that is not shared with *Spry4*.

Previous studies where Sprouty expression is increased or decreased in animal models have shown even more severe craniofacial dysmorphology, including facial clefting associated with a genomic deletion that included *Spry2* (Welsh *et al.* 2007) and cyclopia in a knockout mouse of both *Spry2* and *Spry4* (Taniguchi *et al.* 2007). It is likely that the combined effect of losing multiple factors, either in the chromosomal region surrounding *Spry2* or two Sprouty genes at the same time, directly explains the more severe dysmorphology in these previous models. Although Sprouty loss can lead to reduced growth of the face, overexpression of *Spry1* in the neural crest-derived cells of mice (Yang *et al.* 2010) and overexpression of *Spry2* in the faces of chick embryos (Goodnough *et al.* 2007) can also lead to reduced facial growth and clefting. Because Sprouty genes act as integrators of cell signaling for various growth factors (Guy *et al.* 2009), perhaps even as both agonists and antagonists of the same RTK pathways (Goodnough *et al.* 2007), it is possible that increased and decreased expression of Sprouty can lead to a similar breakdown in regulatory signaling associated with relevant regulatory cascades.

### Sprouty deletion background effects

Previous studies in mice and a variety of other species have indicated that inbred backgrounds can modulate the effects of specific genetic mutations or environmental perturbations. The few results related to changes in morphological shape include significant background by mutation effects on fly wing shape (Dworkin and Gibson 2006), where background effects likely involve genes that are different to those that underlie the main effects of a mutation (Dworkin *et al.* 2009). Systematic differences in background susceptibility of wing shape to multiple mutations that influence *sdE3* expression have been illustrated (Chari and Dworkin 2013). Additionally, chick and zebrafish strains consistently varied in facial shape changes related to early ethanol exposure (Su *et al.* 2001; Loucks and Carvan 2004), including differences in chick ocular diameter and mandible length. In mice, a mutation associated with Treacher Collins syndrome led to widely differing effects on head morphology when placed on different genetic backgrounds. It was proposed that background susceptibility to mutations associated with neural tube defects may be related to normal background variation in the timing and position of neural tube closure (Dixon and Dixon 2004). Whether the phenotype studied is shape or a different quantitative or qualitative trait, there is frequently variation in the susceptibility of genetic backgrounds to developmental and genetic perturbations.

Comparing the severity of Sprouty gene loss across backgrounds, our results suggest that the C57 background is most susceptible to perturbations in Sprouty signaling, while the FVB background is most resilient to these perturbations. It is also possible that variation in genetic background accounts for variation in the penetrance of tooth phenotypes associated with these Sprouty null mutations (Marangoni *et al.* 2015). Understanding why certain backgrounds display higher resilience or opposite phenotypic effects is valuable in the search for genes and genetic pathways associated with variation in the phenotypic results of known disease alleles.

Beyond being more resilient, *Spry1^−/−^* mice on the FVB background actually displayed larger overall skull size, a phenotypic effect opposite to that of Sprouty loss in any other genotype–background combination, including the severe skull size reduction noted in *Spry1^−/−^* mice on the C57 background. We expected to see variation in the severity of a mutation between genotypes, which can be described as a dispersal or spreading of phenotypic outcome across backgrounds. However, the reversed *Spry1^−/−^* FVB size effect represents crossing of phenotypic outcomes between backgrounds, which has previously been noted for gene–gene interactions (de Brito *et al.* 2005) and genotype–environment interactions (Lawson *et al.* 2011). When crossing occurs, differences in genetic background result in opposite phenotypic effects for a given mutation. In cases of crossing, differences in background genotype can have substantial evolutionary consequences (de Brito *et al.* 2005). Hypothetically, natural selection for smaller skulls or larger skulls might both favor the same *Spry1* mutation, depending on whether the population was C57 or FVB.

While the genotype/background combinations that display the most severe dysmorphology tend to display similar overall effects, there are also differences between backgrounds in the specific morphological changes noted. Further exploration of these differences in background effect may be valuable in determining the cellular populations that are being differentially affected by Sprouty loss. For example, the reduced cranial vault length noted in the most severely affected groups may be the result of frontal bone length reduction on the C57 background, which typically has long frontal bones, and parietal bone length reduction on the 129 background, which typically has long parietal bones (Figure 7 and Table 6). In each background, the typically longer bone appears more susceptible to the effects of Sprouty null mutations, which may modify mechanisms of normal formation and maintenance of the coronal suture.

The frontal bone ossifies from a condensation of neural crest-derived mesenchyme, while the parietal bone ossifies from a condensation of mesodermally-derived mesenchyme (Jiang *et al.* 2002). In mice, there is a discrete boundary between these two cell populations at the presumptive coronal suture days before ossification of these bones begins (Jiang *et al.* 2002). It has recently been suggested that the position of the future coronal suture might be determined by the edge of an overlying cartilage called the tectum transversum (Kawasaki and Richtsmeier 2017). During early vault ossification, a discrete sutural zone of unossified mesodermally-derived mesenchyme expressing *Twist1* forms the border between the frontal and parietal bone growth fronts, which express *Fgfr* genes at their edges (Iseki *et al.* 1999; Johnson *et al.* 2000; Rice *et al.* 2000; Morriss-Kay and Wilkie 2005).

Even after intense study of this suture, it is not clear whether some molecular factor predefines the location of this neural crest/mesodermal border or if the border (and associated gene expression patterns) simply occurs wherever the two cell populations meet. If the former is true, variation in the location at which a regulatory factor is expressed might define coronal suture location. In the latter case, variation in speed of cell proliferation and migration of the two preosteogenic populations might define coronal suture location. Either way, Sprouty null mutations appear to modify whatever mechanisms lead to differences in the relative vault bone length of 129 and C57 control mice. We speculate that significant developmental perturbations caused by these mutations may lead to relative vault bone lengths more characteristic of an ancestral middle ground, which C57 and 129 have drifted away from in opposite directions over time. This hypothesized phenotypic convergence may be driven by canalization, which may also explain similarities in direction of skull shape change across first generation hybrids of eight inbred mouse lines (Pavlicev *et al.* 2016).

### Genetic basis of background effects

Inbred background modulates the strength and direction of change in craniofacial size and shape caused by the deletion of Sprouty genes, which are major regulators of RTK pathways. These differences in phenotypic effect are based on epistatic interactions between Sprouty and unknown modifier genes. Moving forward, we want to identify exactly which genes are interacting with Sprouty genes to cause this variation. *Spry2* loss of function on the 129 and C57 backgrounds led to severe reductions in skull size and similar directions of shape change, while these severe effects did not occur on the FVB background. Because only FVB displayed compensation for *Spry2* loss, we expect that the responsible modifier gene(s) should be found in regions of the genome where FVB differs from both 129 and C57.

Because the haplotypes of these inbred strains are >90% identical (Kirby *et al.* 2010), the candidate regions within which relevant modifier genes might be found represent a minority of the genome. Interrogation of published haplotypes in combination with protein interaction databases can provide a solid set of protein coding genes that might underlie FVB background resilience to *Spry2* loss. However, genetic recombination between mutant mice of these inbred backgrounds will allow us to actually map the relevant gene interaction effects to specific genomic regions. Crossing inbred strains of mice with a known disease allele can improve the statistical power of epistatic gene mapping. While a typical search for pairwise epistatic interactions exponentially increases the number of statistical tests required during a genotype–phenotype association study, only a single pass across the genome is necessary if researchers search for modifiers of a single gene of interest (Wei *et al.* 2010). It is likely that this type of one-dimensional screen would be a powerful approach to identify epistatic interactions with a known disease mutation (Mackay 2014).

Crossing mice of different backgrounds allows for recombination between inbred genomes and mapping of genes underlying normal variation to short genomic regions (Darvasi 1998). For example, quantitative trait locus (QTL) analysis of a variety of traits, including skull morphology, has been completed on advanced intercross mice derived from inbred mice of large (LG/J) and small (SM/J) body weights (Leamy *et al.* 1999; Wolf *et al.* 2005; Kenney-Hunt *et al.* 2008). Recombinant inbred strains, such as the Collaborative Cross mice, which are derived from eight genetically diverse inbred lines (Churchill *et al.* 2004; Chesler *et al.* 2008; Collaborative Cross Consortium 2012) or their Diversity Outbred cousins (Svenson *et al.* 2012), can also serve as the basis for relatively fine resolution genotype–phenotype association studies. Characterizing specific mutations on multiple inbred strains and various hybrids can ideally lead to the identification of modifier genes (Linder 2006). For instance, a first generation backcross of mice sensitized for heart failure identified multiple modifier QTL (Le Corvoisier *et al.* 2003). Recently, a series of simple intercrosses and backcrosses between a mutant inbred strain and other inbred strains identified and confirmed a region of chromosome 17 that modifies the expression of a SOD1 mutation linked to amyotrophic lateral sclerosis (Sher *et al.* 2014). Increasing the amount of recombination between these types of inbred mutant lines through further intercrossing, backcrossing, or other breeding strategies will yield mouse populations with better genomic resolution for modifier gene identification, as we see with advanced intercross lines and recombinant inbred strains (Jarvis and Cheverud 2011; Collaborative Cross Consortium 2012; Svenson *et al.* 2012).

Advanced crosses of multiple strains with the same deleterious mutation have been completed successfully in other animal models. For example, intercrossing independent fly lines sharing a mutant allele, followed by backcrossing those that retained the associated phenotype of interest, led to independent lines for use in modifier gene mapping (Chandler *et al.* 2014). Hybrid crosses of yeast strains with mutations that displayed a lethal phenotype in a subset of those strains indicated that multiple modifier genes are likely associated with the noted strain-specific genetic variation (Dowell *et al.* 2010). Introgression of recessive alleles onto various wild isolates of *Caenorhabditis elegans* showed that the effects of mutations known to influence vulval cell fate varied significantly between genetic backgrounds (Milloz *et al.* 2008). QTL analysis of sixty recombinant inbred lines derived from two of these introgressed lines identified genomic regions, including a specific polymorphism, that partially explain background effects on vulval cell fate (Duveau and Félix 2012). The results of these and similar studies in other model organisms illustrate the potential value of careful and diverse breeding programs to identify unknown epistatic interactions that underlie variation in mutant trait penetrance and severity.

Mouse models are, of course, associated with longer generation times and increased husbandry expenses, which may be exacerbated if offspring carrying a mutation are less viable. While identifying novel epistatic interactions with certain disease-associated mutations may be worth the current expense of a long-term mouse breeding program, it is possible that technological advances will make searching for epistatic effects in mice more practical in the near future. For instance, as mouse CRISPR technology becomes more commonly available and efficient (Markossian and Flamant 2016; Tschaharganeh *et al.* 2016), it should be possible to delete a gene or introduce a mutation of interest onto existing recombinant inbred strains, like the Collaborative Cross mice. Alternatively, it may not always be necessary to introduce the mutation of interest onto all inbred backgrounds under study. Recently, a complex series of intercrosses and backcrosses between a mutant inbred strain and other inbred strains identified and confirmed a region of chromosome 17 that modifies the expression of a SOD1 mutation linked to amyotrophic lateral sclerosis (Sher *et al.* 2014). The breadth of strategies for the analysis of epistatic interactions is reviewed by Mackay (2014).

### Conclusions

Our results show that significant mutant genotype-by-background interactions (epistatic interactions) exist in mouse models and support the notion that mutations must be characterized on multiple genetic backgrounds in order to fully understand the phenotypic effects of those mutations (Linder 2006). Strong epistatic effects on complex traits, such as those quantified here, are likely common across mouse models and human populations. In fact, epistasis may account for some of the phenotypic variance that is not explained by single locus additive genetic effects (Mackay 2014), a phenomenon known as “missing heritability.” Intercrossing Sprouty null mice from multiple backgrounds or the use of other breeding strategies may allow for the identification of previously unknown genetic interactions that modulate the effect of Sprouty alleles on a variety of phenotypes. Accounting for epistatic interactions like those documented here is necessary to gain a deeper understanding of the genotype–phenotype map and to explain a significant proportion of the variation in adult phenotypes and genetic disease severity.

## Supplementary Material

Supplemental material is available online at www.g3journal.org/lookup/suppl/doi:10.1534/g3.117.040659/-/DC1.
