# Supplementary material for: The Interaction of Genetic Background and Mutational Effects in Regulation of Mouse Craniofacial Shape
Source: G3 (Bethesda). 2017 Mar 8;7(5):1439–50. doi: 10.1534/g3.117.040659 (PMC5427488; doi:10.1534/g3.117.040659)
Supplement: Supplementary file 1 [file 1439FileS1.docx]

**Materials and Methods Supplement**

Mouse Breeding

All mice (*Mus musculus*) were bred at the University of California, San Francisco in compliance with relevant animal care guidelines and experimentation protocols. Mouse lines carrying null alleles of *Spry1* (Basson *et al.* 2005), *Spry2* (Shim *et al.* 2005), and *Spry4* (Klein *et al.* 2006) were acquired as knockout mice (gift from Gail Martin) and maintained on a Crl:CD1 mixed background within the Klein lab. These three strains were then independently backcrossed, using the standard procedure for producing congenic lines, onto the 129X1/SvJ (RRID:IMSR_JAX:000691), C57BL/6J (RRID:IMSR_JAX:000664), and FVB/NJ (RRID:IMSR_JAX:001800) backgrounds for between 6 and 27 generations (See File S3 for specimen generation numbers). After a first generation cross between KO and inbred background mouse, congenic backcrossing is defined as crossing a partially inbred heterozygote that has the Sprouty mutation of interest with an inbred background mouse. Heterozygotes of these backcrossed null mice were crossed to produce litters that include homozygote null (-/-), heterozygote null (+/-), homozygote controls (+/+) of each knockout by background pair. Therefore, our sample includes representatives from 18 genotype by background combinations (Table 1). While some of our sample were not strictly isogenic in their genetic background, congenic backcrossing for 6 generations results in > 95% homozygosity across the genome (Silver 1995). All specimens were sacrificed at approximately eight weeks of age and stored at -20°C.

The three inbred mouse lines were acquired from Jackson Laboratories who use a Genetic Stability Program to limit cumulative genetic drift in both the C57BL/6J and FVB/NJ by rebuilding foundation stocks from cryopreserved embryos every five generations (<https://www.jax.org/jax-mice-and-services/find-and-order-jax-mice/why-jax-mice/patented-genetic-stability-program>). The isogenecity of the 129X1/SvJ is maintained through more traditional methods of careful colony management and periodic genotyping to identify genetic contamination.

During the process of backcrossing, the Sprouty genotype of each specimen was assessed using PCR. *Spry1* genotyping was performed using a trio of specific primers (*Spry1 71*-CTC AAT AGG AGT GGA CTG TGA AAC TGC; *Spry1 72*-GGG AAA ACC GTG TTC TAA GGA GTA GC; *Spry1 73*-GTT CTT TGT GGC AGA CAC TCT TCA TTC) detecting the WT allele (311 bp, amplicon *71/72*) or the null allele (150 bp, amplicon *71/73*), with the following steps: 5 min at 95°C, 33 cycles of 30 s at 95°C-30 s at 60.8°C-45 s at 72°C, and 5 min at 72°C. *Spry2* genotyping was performed using a trio of specific primers (*Spry2 GM148*-TTG AGA ACA TGC CTC GAC C; *Spry2 GM138*-GCA TGG GCT ATT CAC AAA C; *Spry2 GM12*-GGA TGG CTC TGA TCT GAT CC) detecting the WT allele (350 bp, amplicon *148/12*) or the null allele (250 bp, amplicon *148/138*), with the following steps: 10 min at 95°C, 35 cycles of 40 s at 45 s at 50°C-1 min at 72°C, and 5 min at 72°C. *Spry4* genotyping was performed using a trio of specific primers (*Spry4 F1*-CAG GAC TTG GGA GTG CTT CCT TAG; *Spry4 B3*-CCT CCT AGT ACC TTT TTG GGG AGA G; *Spry4 B4*-TAC AGC AGG AAT GGC TAC GGT G) detecting the WT allele (300 bp, amplicon *F4/B3*) or the null allele (459 bp, amplicon *B3/B4*), with the following steps: 10 min at 95°C, 31 cycles of 1 s at 94°C-1 min at 63°C-1 min at 72°C, and 5 min at 72°C. Further details about the PCR primers can be found in supplementary file S4.

Skull Measurement

Micro-computed tomography images of specimen heads were acquired with a Scanco vivaCT 40 at the University of Calgary with 55kV/145µA for images of 0.035 mm^3^ voxel size. Three dimensional coordinates of 54 previously defined (Percival *et al.* 2016) adult landmarks (8 midline, 46 bilateral) were collected by a single observer from minimum threshold defined bone surfaces within Analyze 3D (www.mayo.edu/bir/). These landmarks display low repeatability error and were chosen to represent overall morphology of the skull at a moderate landmark density.

We completed two general categories of morphometric analysis: 1) Euclidian Distance Matrix Analysis (EDMA) and 2) Procrustes superimposition based Geometric Morphometrics. EDMA methods are based on ratios of linear distances calculated between skull landmarks for pairs of genotypes (Lele and Richtsmeier 2001). A given linear distance differs between two genotypes when the linear distance ratio is significantly different than one. These methods allow us to visualize raw differences in unscaled form (the combination of size and shape) between pairs of genotypes and to identify specific regions of the skull that increase or decrease in size when a Sprouty gene is knocked out. EDMA analyses were completed with custom functions in R (R Developmental Core Team 2008), which were based on published formulas (Lele and Richtsmeier 2001) and R code (Hu 2007).

We also completed Procrustes superimposition of specimens, which rotates, translates, and scales each specimen’s landmark coordinates into the same space (Zelditch *et al.* 2012). Geometric morphometric analyses based on the resulting Procrustes coordinates allow us to compare broader patterns of craniofacial shape variation between genotypes after removing differences in scale, as estimated by centroid size of each specimen’s landmark constellations. Procrustes superimposition, calculation of centroid sizes, and Procrustes ANOVA were performed using the geomorph package (Adams and Otárola-Castillo 2013) in R.

Size Effects

Significant differences in mean centroid size between homozygote knockout specimens and their littermate hybrid controls were identified by comparing knockout mean values with a 95% confidence interval that was produced with bootstrap tests (1000 permutations) of control centroid size values. EDMA FORM analysis was used to identify linear distances that significantly differ between homozygote knockouts and their littermate controls (α=0.05). Histograms of all EDMA linear distance ratio estimates, colored by significance, were produced to visualize the severity and extent of size changes across the skull for each pair of genotypes.

Shape Effects

ANOVA of Procrustes superimposed coordinates was completed for all specimens in our sample, with genotype (e.g. *Spry1*^+/-^), inbred background (e.g. C57), and the interaction between genotype and inbred background as covariates. This analysis was used to determine whether these genetic covariates contribute significantly to adult skull shape and how much of the variance in skull shape they are associated with. Given that inbred background had the largest effect on skull shape, we calculated the Procrustes distances between mean controls of inbred background pairs. Procrustes distances are calculated as the sum of distances between corresponding landmarks of two shapes after Procrustes superimposition. A bootstrapping algorithm was used to determine if the Procrustes distance values were significantly different than 0 (α=0.05). In order to visualize the typical shape differences between inbred backgrounds, we plotted the mean Procrustes coordinates of each background, arbitrarily using the 129 background as the basis for comparison.

Given that Sprouty genotype also has a significant effect on skull shape, we calculated Procrustes distances between homozygous deletions (-/-) and homozygous controls (+/+) of each genotype-background combination, followed by a visualization of differences between mean Procrustes coordinates of these pairs to identify the shape differences associated with Sprouty loss of function. The results of Procrustes-superimposition based geometric morphometrics illustrated relative differences in shape between genotypes.

Correlations between vectors of shape change quantified whether the direction of skull shape change is similar when a given null mutation is found on different backgrounds. Vector correlations were calculated from mean shape differences as quantified from principal component scores, similar to the way vector correlations are calculated in trajectory.analysis() within geomorph (Collyer and Adams 2013). Permutations (n=1000) of vector correlations calculated from four groups of 20 random specimens were used to produce a 95% confidence interval to determine whether vector correlations are significantly different than zero.

To further explore the effects of Sprouty loss of function on specific morphological features of interest, we compared ratios of specific linear distances that were generated during EDMA FORM analysis. For instance, we explored whether the relatively short and wide skull of strongly affected genotypes was the result of a reduction in raw skull length and an increase in raw skull width or this shape change was the result of a less severe reduction in skull width compared to length. We also compared the ratios of linear distances between midline vault landmarks to investigate whether reduced length of specific midline bones leads to the shorter vault lengths noted in Sprouty null mice with the most extreme dysmorphology.
