# Supplementary material for: The Interaction of Genetic Background and Mutational Effects in Regulation of Mouse Craniofacial Shape
Source: G3 (Bethesda). 2017 Mar 8;7(5):1439–50. doi: 10.1534/g3.117.040659 (PMC5427488; doi:10.1534/g3.117.040659)
Supplement: Supplementary file 4 [file 1439FileS4.docx]

|  | Primer Name | Primer Sequence |
| --- | --- | --- |
| Primer A | Spry1 71 | CTC AAT AGG AGT GGA CTG TGA AAC TGC |
| Primer B | Spry1 72 | GGG AAA ACC GTG TTC TAA GGA GTA GC |
| Primer C | Spry1 73 | GTT CTT TGT GGC AGA CAC TCT TCA TTC |
|  |  |  |
|  | Length | Gene Detected |
| Amplicon A/B | 311 b.p. | Wildtype |
| Amplicon A/B | ~340 b.p. | Floxed Allele |
| Amplicon A/C | 150 b.p. | Null |

|  | Thermocycler Prgm. | |
| --- | --- | --- |
| Folder | CIP |  |
| Name | Spry1 |  |
| Hot-start | 95C for 5:00 |  |
| Denature | 95C for 0:30 | Repeat 33X |
| Anneal | 60.8C for 0:30 |  |
| Elongation | 72C for 0:45 |  |
| Final Elong. | 72C for 5:00 |  |
| Hold | 4C for ever |  |

|  | Primer Name | Primer Sequence |
| --- | --- | --- |
| Primer A | Spry2 GM148 | TTG AGA ACA TGC CTC GAC C |
| Primer B | Spry2 GM138 | GCA TGG GCT ATT CAC AAA C |
| Primer C | Spry2 GM12 | GGA TGG CTC TGA TCT GAT CC |
|  |  |  |
|  | Length | Gene Detected |
| Amplicon A/B | 250 | Knocked out Spry2 |
| Amplicon A/C | 350 | Wildtype Spry2 |

|  | Thermocycler Prgm. |  |
| --- | --- | --- |
| Folder | AJ |  |
| Name | Spry --> Spry2 |  |
| Hot-start | 95C for 10:00 |  |
| Denature | 95C for 0:40 | Repeat 35X |
| Anneal | 50C for 0:45 |  |
| Elongation | 72C for 1:00 |  |
| Final Elong. | 72C for 5:00 |  |
| Hold | 4C for ever |  |

|  | Primer Name | Primer Sequence |
| --- | --- | --- |
| Primer A | Spry4 F1 | CAG GAC TTG GGA GTG CTT CCT TAG |
| Primer B | Spry4 B3 | CCT CCT AGT ACC TTT TTG GGG AGA G |
| Primer C | Spry4 B4 | TAC AGC AGG AAT GGC TAC GGT G |
|  |  |  |
|  | Length | Gene Detected |
| Amplicon A/B | 300 | Wiltype Spry4 |
| Amplicon A/B | 425 | Floxed Spry4 |
| Amplicon A/C | 459 | Knocked out Spry4 |

|  | Thermocycler Prgm. | |
| --- | --- | --- |
| Folder | AJ |  |
| Name | Spry --> S4-57 |  |
| Hot-start | 95C for 10:00 |  |
| Denature | 94C for 1:00 | Repeat 31X |
| Anneal | 63C for 1:00 |  |
| Elongation | 72C for 1:00 |  |
| Final Elong. | 72C for 5:00 |  |
| Hold | 4C for ever |  |
